# Effect of Sex, Anesthesia Method, Physiological Asymmetry, and Strain on Rat Lymphatic Vessel Contractility Assessed via Near‐Infrared Spectroscopy and Linear Mixed‐Effects Modeling

**DOI:** 10.1111/micc.70087

**Published:** 2026-09-11

**Authors:** Sophia Mavris, Rachel Chin, Shao‐Yun Hsu, Rudolph L. Gleason, J. Brandon Dixon, Zhanna Nepiyushchikh

**Affiliations:** ^1^ The Wallace H. Coulter Department of Biomedical Engineering 313 Ferst Dr., Georgia Institute of Technology and Emory University Atlanta Georgia USA; ^2^ The George W. Woodruff School of Mechanical Engineering 801 Ferst Dr., Georgia Institute of Technology Atlanta Georgia USA; ^3^ The Parker H. Petit Institute for Bioengineering and Bioscience 315 Ferst Dr., Georgia Institute of Technology Atlanta Georgia USA

**Keywords:** anesthesia, linear mixed‐effects modeling, lymphatic contractility, lymphatic physiology, lymphatic transport, lymphedema

## Abstract

**Objective:**

This study aims to investigate the effect of sex, anesthesia administration method, lymphatic physiological asymmetry, and strain on popliteal lymphatic collecting vessel contractile function and transport in effort to create a research comparison standard for experimentations using rat popliteal lymphatics.

**Methods:**

Naïve female and male (8–10 weeks old) Sprague–Dawley and Lewis rats (both left and right hind limbs) under two forms of anesthesia administration (ketathesia/dexdomitor and isoflurane) were used to assess lymphatic contractility and transport function via near‐infrared imaging and linear mixed‐effects modeling.

**Results:**

We quantified lymphatic contractility and transport through five functional metrics (packet frequency, packet amplitude, normalized packet transport, packet width, and normalized packet integral) via near‐infrared imaging and found that both sex and anesthesia method significantly affect pumping behavior, while physiological asymmetry had minimal influence. Additionally, we identified strain‐dependent differences in contractile dynamics between Sprague Dawley and Lewis rats. Linear mixed‐effects modeling further supported these results, highlighting the significant and differential roles that sex, anesthesia method, and strain play in modulating lymphatic contractile function.

**Conclusions:**

Our results showcase that to create standard comparison criteria for rat popliteal lymphatic models, one consistent method of anesthesia is essential and for sex to be considered as a biological variable. These findings underscore the physiological variability that must be accounted for when modeling lymphatic function and disease.

**Abbreviations**
CLV: collecting lymphatic vessel
G&R: growth and remodeling
LEC: lymphatic endothelial cell
LMC: lymphatic muscle cell
NIR: near‐infrared spectroscopy
NO: nitric oxide
ROI: region of interest
SD: standard deviation
SEM: standard error of the mean

## 1 Introduction

The lymphatic system plays a crucial role in maintaining fluid homeostasis by absorbing interstitial fluid and transporting it back to the cardiovascular system [1, 2]. This transport relies heavily on the proper contractile behavior of the collecting lymphatic vessels (CLVs), which drive lymph propulsion through robust phasic and tonic contractions. Unique structural features of the CLVs, such as intraluminal valves that prevent fluid backflow and also a contractile layer of both lymphatic muscle cells (LMCs) and lymphatic endothelial cells (LECs), enable this function [3]. When contractility is impaired due to injury or dysfunction, fluid accumulation in the interstitium can lead to the development of lymphedema [4]. Although the lymphatic system exhibits intrinsic ability to often grow, remodel, and adapt to restore proper lymphatic function (growth and remodeling) through both contractile and structural adaptations, substantial injury to the vasculature can disrupt this process [5, 6, 7]. In such, no cure exists for lymphedema, underscoring the need for more comprehensive understanding of these mechanisms and for novel and effective treatments [8, 9].

Animal models, particularly rats, are widely used to study lymphatic physiology and injury responses. However, differences in model implementation, such as anesthesia method (e.g., ketamine/dexdomitor‐based vs. isoflurane‐based), can introduce variability and limit comparability of findings across studies [10].

The lymphatic fluid, lymph, ultimately reenters the cardiovascular system via two major ducts: the right lymphatic duct and the thoracic duct. The right lymphatic duct empties into the right subclavian veins and drains the majority of lymph from the right upper quadrant of the body, while the thoracic duct empties into the left subclavian veins and has a higher fluid demand as it drains the rest of lymph of the body [11, 12]. This inherent physiological asymmetry in lymphatic drainage raises the possibility that CLV function and contractility may differ between sides of the body.

The LEC layer within CLVs is highly regulated by chromosomal and hormonal sex differences, which play an important role in many physiological functions and have been shown to have an effect on the vascular endothelium, such as cell proliferation, migration, and vascular remodeling [13, 14]. For example, the sex hormone estrogen when bound to the estrogen receptors α and β expressed on LECs and LMCs can affect vasodilation and repair and remodeling processes [15]. Prior studies have reported lower lymphatic pressure in females compared to males [16]; however, comprehensive insight into sex‐related differences in lymphatic function, particularly within the rat popliteal lymphatic region, remains lacking [17]. Addressing this gap could help inform hormonal‐based interventions for lymphatic diseases [17], which are notably more prevalent in women following breast or gynecological cancer treatments [18, 19].

Experimental variables, such as temperature, body positioning, and the use of dyes, are known to impact vessel contractility [20, 21, 22, 23, 24]. However, the specific effects of anesthesia methods on rat lymphatic function are not fully defined. Emerging data from mouse models suggest that isoflurane and pentobarbital inhibit lymphatic contractility, while ketamine‐based anesthesia may preserve it [25]. Establishing an anesthetic standard in rat lymphatic models is critical for ensuring data consistency and for accurately linking molecular mechanisms to observed contractile behaviors [26].

In addition to sex and anesthesia, rat strain can also influence lymphatic function. While Sprague Dawley rats are most used, other strains are selected based on surgical and experimental applications. For example, Wistar rats may show reduced neuropathic pain responses, while inbred Lewis rats offer advantages for transplant procedures [27, 28, 29]. However, perhaps physiological differences across strain may extend to lymphatic contractility.

Lymphatic injury alters CLV contractility and overall transport function through changes in the local biomechanical environment, including interstitial stress, fluid shear stress, and wall tension [30, 31, 32]. Consequently, increased fluid loads elicit adaptive responses in the vasculature, such as increased contraction frequency and decreased tone [33, 34, 35]. LECs sense changes in local fluid shear stress and signal to LMCs to modulate contractility by releasing nitric oxide (NO) as a means of a vasodilation response [36, 37]. In this work, we examine the effects of sex, anesthesia methods, physiological asymmetry, and strain on CLV pumping function in a naïve rat popliteal lymphatic model to provide an essential baseline for future lymphatic injury and lymphedema studies [27, 38, 39, 40, 41]. Furthermore, we compare pumping function metrics between Sprague Dawley and Lewis rats. Linear mixed‐effect modeling is employed to quantify the influence of these factors on CLV and lymph transport.

## 2 Materials & Methods

**FIGURE 1:**
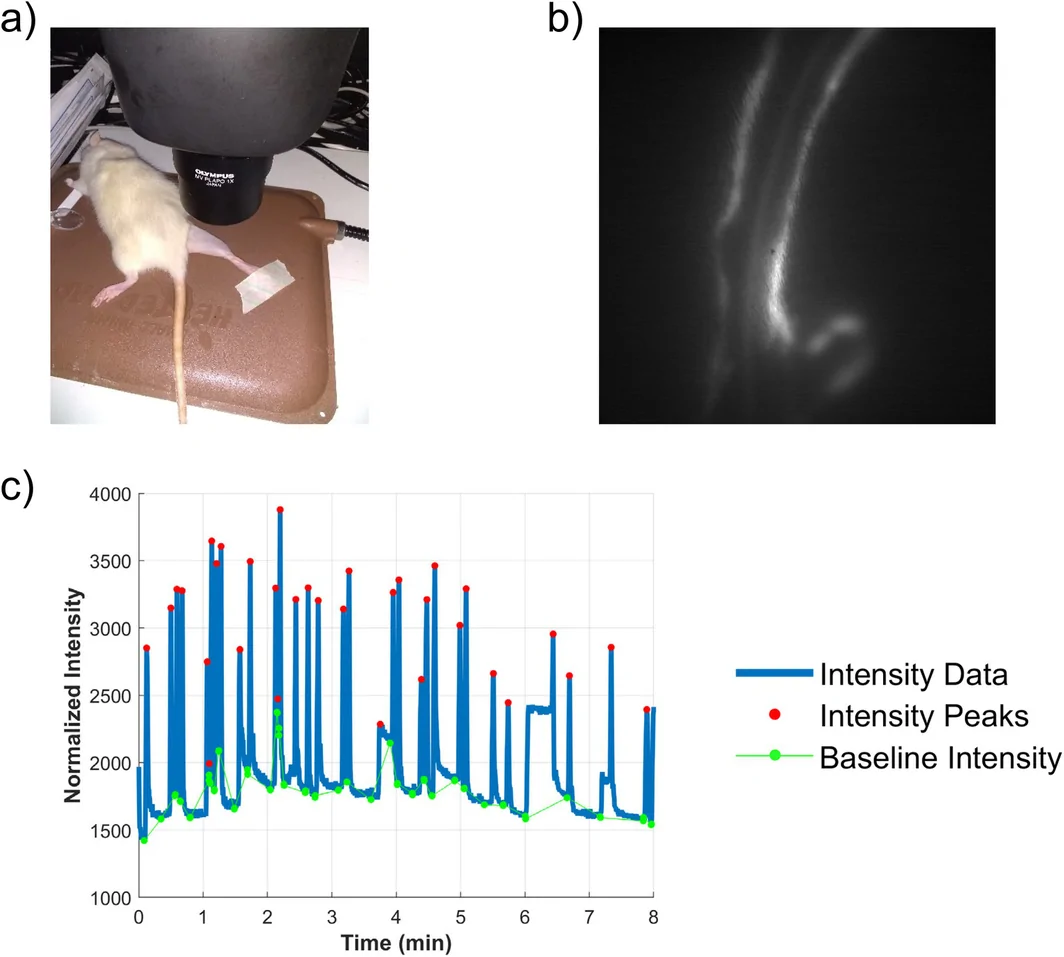
In vivo lymphatic pumping assessment using near‐infrared (NIR) imaging. (a) Rat positioned prone with the hindlimb secured such that the popliteal region is aligned beneath the microscope objective for imaging. (b) Representative NIR image showing two parallel collecting lymphatic vessels within the popliteal region. (c) Normalized fluorescence intensity over time (blue) for a representative near‐infrared (NIR) imaging session acquired at 10 frames per second. Intensity peaks, marked in red, indicate individual contractile events, while green markers represent the interpolated baseline intensity between minima. This fluorescence intensity‐time profile is used to quantify five key metrics of lymphatic pumping function: Packet frequency, amplitude, transport, width, and integral.

A total of 81 rats were included in the study, comprising 18 males and 63 females. We used near‐infrared (NIR) imaging as a technique to measure differences in CLV contractile function via a series of lymphatic transport metrics, such as packet frequency, amplitude, transport, width, and integral. Prior to imaging, each rat was weighed and administered its respective method of anesthesia. For ketathesia/dexdomitor administration, each rat received its respective dosage of 100 mg/mL ketathesia (40 mg/kg) and 0.5 mg/mL dexdomitor (0.13 mg/kg) for sedation by intramuscular (IM) injection at the indicated doses. For isoflurane administration, each rat was placed in a small‐animal chamber with an inward flow of 5% isoflurane gas. Once the rat became initially anesthetized, it was removed from the chamber and placed in a nose cone with a lowered inward flow of 2% isoflurane gas for the duration of imaging. The hindlimb of each rat was shaved to avoid interference with imaging resolution. Each rat was placed on a heating pad in the prone position underneath the Olympus MV PLAPO 1× microscope. 15 μL of a lymphatic tracer (LI‐COR IRDye 800CW NHS Ester conjugated to methoxypolyethylene glycol amine and reconstitution in saline) was injected in the footpad of each rat in between the fourth and fifth digits. In conjunction with the microscope (800 λ), the NIR system consisted of a Lambda LS illuminator, a Lambda Se high‐speed shutter controller, an Evolve Delta camera, and the Micro‐Manager 1.4.22 software. After a waiting period of approximately 5 min for the lymphatics to uptake the tracer, a multi‐dimensional acquisition was taken at an interval of 100 ms and an image quantity of 5000. This process was repeated as both hindlimbs of each rat were imaged (Figure 1a). The imaging order was randomized. For anesthesia reversal, animals who received ketathesia/dexdomitor were awakened with 5 mg/mL of antisedan (1.3 mg/kg), while animals who received isoflurane were removed from the nose cone.

Image sequences acquired using the imaging software were imported into ImageJ to visualize temporal changes in fluorescence intensity [42]. For each rat, two‐to‐four regions of interest (ROIs) were manually drawn over the lymphatic vessels to capture dynamic fluorescence fluctuations. The intensity profiles were then processed to extract five quantitative metrics of lymphatic pumping function. Raw intensity traces were analyzed using a custom peak detection algorithm with a defined threshold to identify significant contractile events, marked by local maxima (peaks) and minima (Figure 1c). Each peak corresponded to a discrete lymphatic contraction, bounded by adjacent minima that defined the temporal extent of the contraction packet. Packet frequency was calculated as the number of detected peaks per minute, based on a frame rate of 10 frames per second. Packet width was defined as the duration (in frames) between the two bounding minima of each contraction. Contraction amplitude was determined as the difference between the peak intensity and a local linear baseline interpolated between the bounding minima. The area under the curve above this baseline defined the packet integral, representing flow output per contraction, while the sum of all packet integrals over time yielded the transport metric, or flow output per unit time. To enable comparisons across experimental conditions, packet amplitude, integral, and transport values were normalized to the local baseline intensity. The NIR imaging and analysis methods used in this study were adapted from previously established studies [43, 44, 45, 46]. A summary of each pumping function metric, including its physiological interpretation and units, is provided in Table 1.

**TABLE 1:** Quantitative metrics of lymphatic vessel contractility derived from intensity‐time analysis.

Metric	Physiological interpretation	Units
Packet frequency	Number of contractions per minute	Contractions/min
Packet width	Average time of systolic peaks within the contraction cycle	Frames
Normalized packet amplitude	Relative strength of contraction	Unitless
Normalized packet transport	Normalized flow output per unit time	Unitless
Normalized packet integral	Normalized flow output per contraction	Unitless

## 3 Results

In this study, we investigated how sex, anesthesia method, physiological asymmetry, and strain affect lymphatic pumping function. Contractility of CLVs was assessed using five pumping metrics: packet frequency, amplitude, transport, width, and integral. Specifically, packet frequency reflects the rate of spontaneous contractions, while packet amplitude and width describe the strength and duration of each contraction, respectively. Packet transport captures the peak lymph flow generated per contraction, and packet integral represents the total lymph volume moved during each contraction cycle.

**FIGURE 2:**
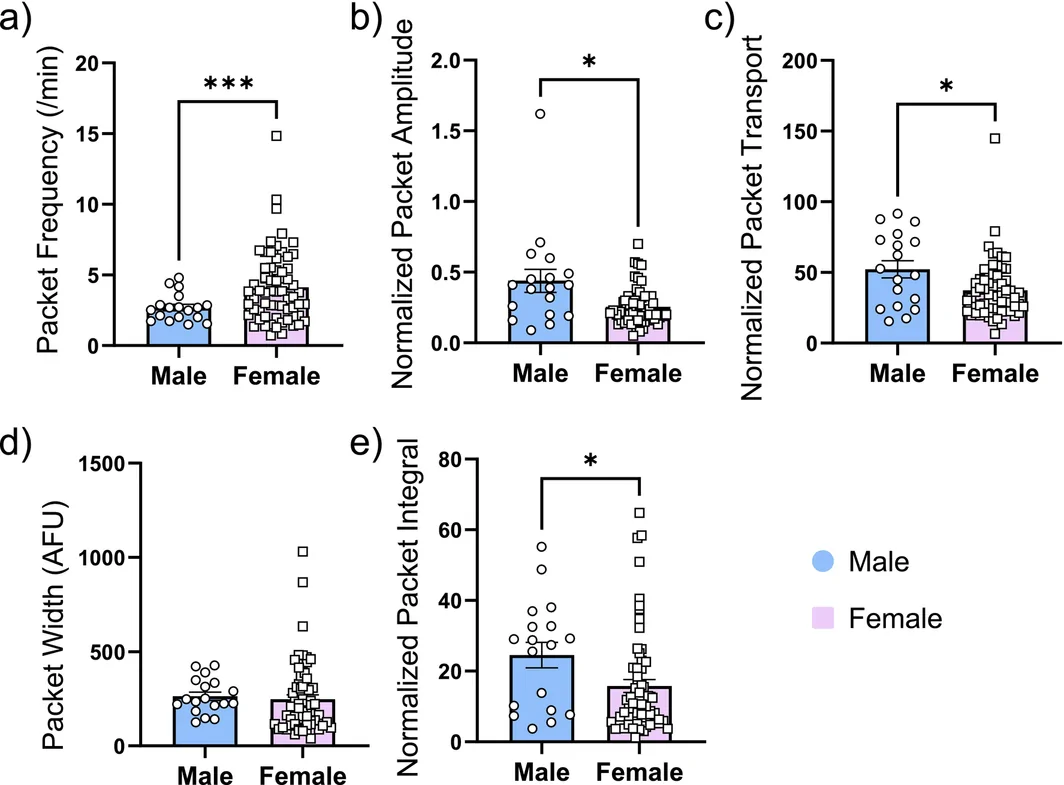
Sex‐based differences in lymphatic pumping metrics between male (blue, circle) and female (purple, square) Sprague Dawley rats. Quantified metrics include (a) packet frequency (/min), (b) normalized packet amplitude, (c) normalized packet transport, (d) packet width (frames), and (e) normalized packet integral. Each data point represents an individual sample, with error bars denoting the standard error of the mean (SEM). Solid lines above data points indicate statistically significant pairwise comparisons based on unpaired *t*‐tests with Welch's correction (* = *p* < 0.05, ** = *p* < 0.01, and *** = *p* < 0.001, *n* = 18 males, *n* = 63 females).

We first compared male and female Sprague Dawley rats, combining data across hindlimbs and anesthesia methods (Figure 2). Female rats exhibited significantly lower packet amplitude, transport, and integral, but a higher packet frequency compared to males. No significant differences in packet width were observed between sexes.

**FIGURE 3:**
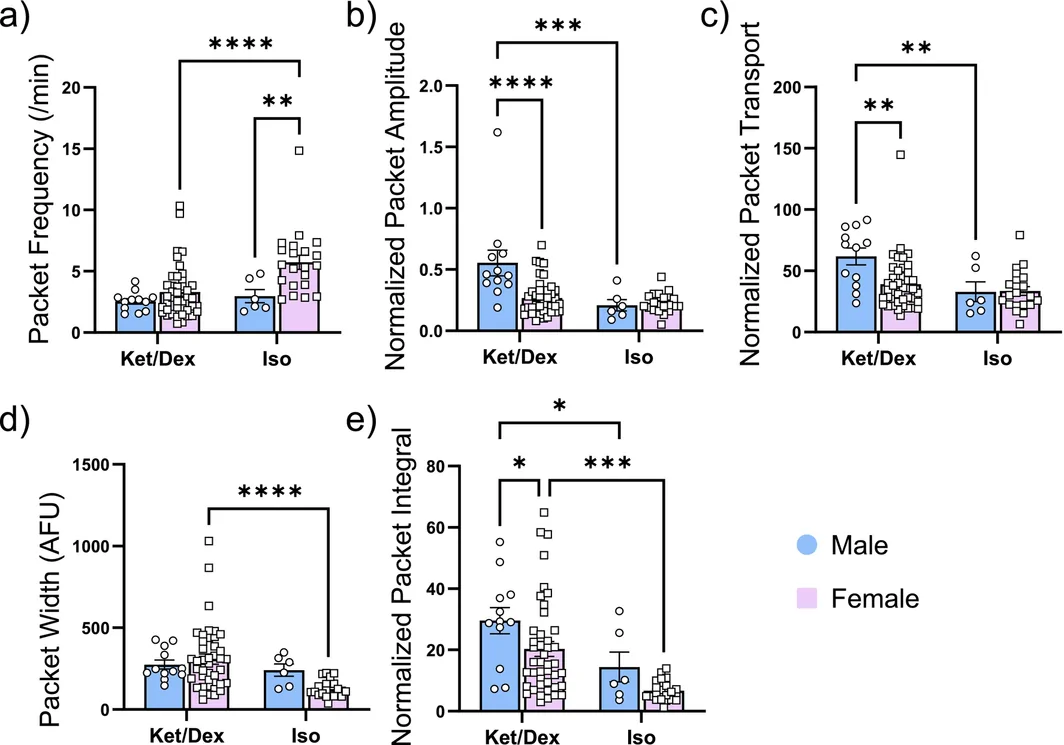
Effect of anesthesia type (ketamine/dexdomitor and isoflurane) on lymphatic pumping metrics in male (blue, circle) and female (purple, square) Sprague Dawley rats. Quantified metrics include (a) packet frequency (/min), (b) normalized packet amplitude, (c) normalized packet transport, (d) packet width (frames), and (e) normalized packet integral. Each data point represents an individual sample, with error bars denoting the standard error of the mean (SEM). Solid lines above data points indicate statistically significant pairwise comparisons based on a two‐way ANOVA with multiple comparisons correction (* = *p* < 0.05, ** = *p* < 0.01, *** = *p* < 0.001, and **** = *p* < 0.0001, *n* = 12 males, Ket/Dex, *n* = 42 females, Ket/Dex, *n* = 6 males, Iso, *n* = 21 females, Iso).

Next, we examined the effect of anesthesia method (i.e., comparing ketamine/dexdomitor to isoflurane) with sex‐stratified groups (Figure 3). Among female Sprague Dawley rats, isoflurane anesthesia significantly increased packet frequency but reduced packet width and integral relative to ketamine/dexdomitor. Among male Sprague Dawley rats, isoflurane significantly reduced packet amplitude, transport, and integral compared to ketamine/dexdomitor. Additionally, isoflurane yielded higher packet frequency in female rats compared to males, while ketamine/dexdomitor led to higher packet amplitude, transport, and integral in males compared to females.

**FIGURE 4:**
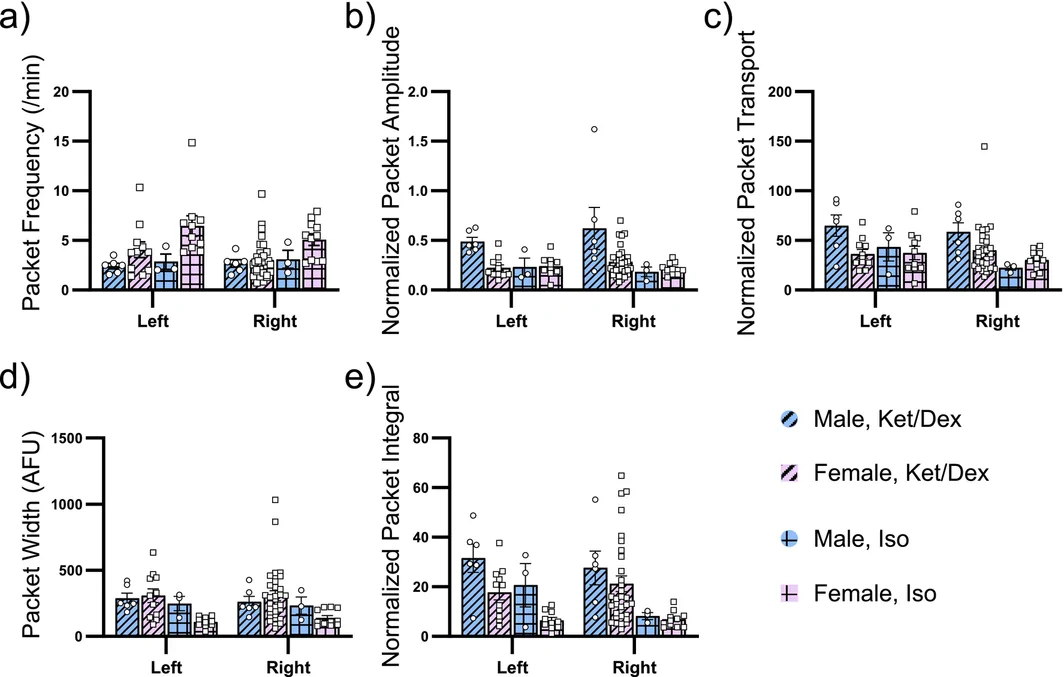
Effect of physiological asymmetry (left and right hindlimbs) on lymphatic pumping metrics across sex and anesthesia conditions. Groups include male (blue, circle) and female (purple, square) Sprague Dawley rats anesthetized with either ketamine/dexdomitor (diagonal) or isoflurane (grid). Quantified metrics include (a) packet frequency (/min), (b) normalized packet amplitude, (c) normalized packet transport, (d) packet width (frames), and (e) normalized packet integral. Each data point represents an individual sample, and statistical significance between groups was assessed using a two‐way ANOVA with Tukey's multiple comparisons correction (*n* = 6 males, Ket/Dex, left, *n* = 11, females, Ket/Dex, left, *n* = 3 males, Iso, left, *n* = 10 females, Iso, left; *n* = 6 males, Ket/Dex, right, *n* = 31 females, Ket/Dex, right, *n* = 3 males, Iso, right, *n* = 11 females, Iso, right).

To assess physiological asymmetry, we compared lymphatic pumping metrics between left and right hindlimbs, stratified by both sex and anesthesia method (Figure 4). No significant differences were observed between limbs across any group, indicating minimal physiological asymmetry in pumping function under the tested conditions.

**FIGURE 5:**
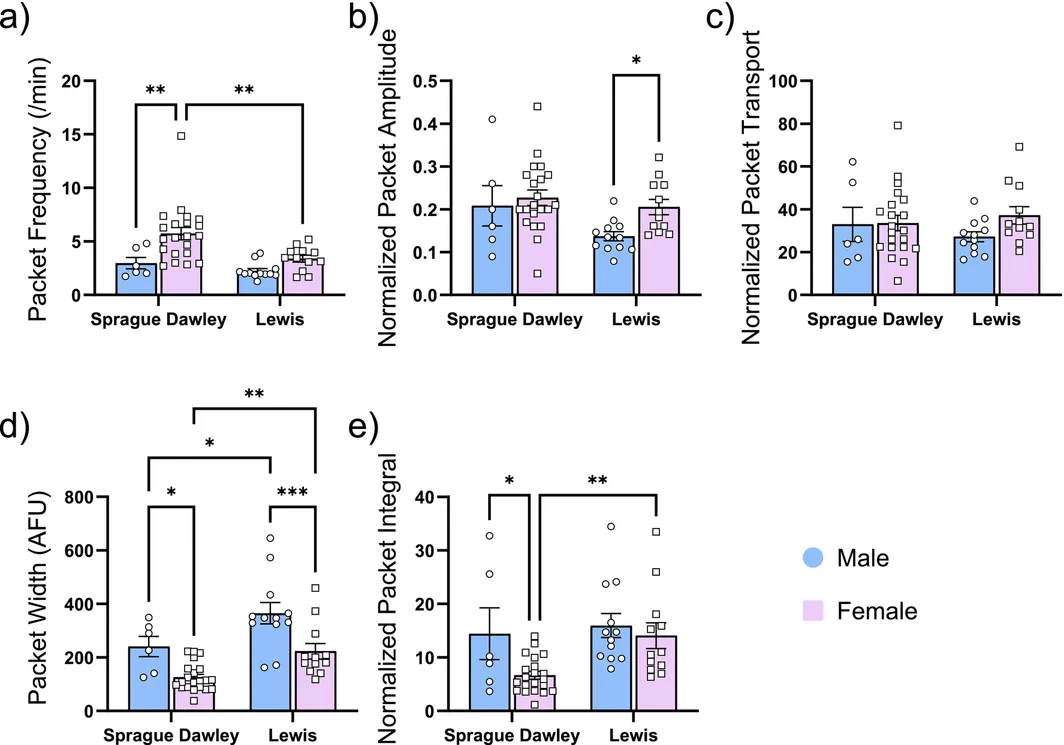
Strain (Sprague Dawley vs. Lewis) comparison of lymphatic pumping metrics in male (blue, circle) and female (purple, square) rats under isoflurane anesthesia. Quantified metrics include (a) packet frequency (/min), (b) normalized packet amplitude, (c) normalized packet transport, (d) packet width (frames), and (e) normalized packet integral. Each data point represents an individual sample, with error bars denoting the standard error of the mean (SEM). Solid lines above data points indicate statistically significant pairwise comparisons based on a two‐way ANOVA with multiple comparisons correction (* = *p* < 0.05, ** = *p* < 0.01, and *** = *p* < 0.001, *n* = 6 males, Sprague–Dawley, *n* = 21 females, Sprague–Dawley, *n* = 12, males, Lewis, *n* = 12, females, Lewis).

We then compared rat strain, evaluating differences between Sprague Dawley and Lewis rats under isoflurane anesthesia, stratified by sex and combining limbs (Figure 5). Among females, Lewis rats exhibited lower packet frequency and higher packet width and integral compared to Sprague Dawley rats. Among males, Lewis rats showed increased packet width, but other metrics were comparable between strains.

To evaluate the relative influence of each factor on lymphatic function, we applied a linear mixed‐effects model using MATLAB's “fitlme” function. Fixed effects included strain, sex, anesthesia method, and limb side, while rat ID was modeled as a random effect. The model produced coefficient estimates (β) for each factor and metric. These coefficients represent the difference in outcome between the compared and reference groups (as listed in Table 2). A positive β indicates the compared group has a higher value than the reference group, while a negative β indicates a lower value. The magnitude of β reflects the strength of that factor's impact.

**TABLE 2:** Linear mixed‐effects model reference and compared groups.

Predictor	Reference group	Compared group
Sex	Female	Male
Anesthesia	Isoflurane	Ketamine/Dexdomitor
Leg	Left	Right
Strain	Lewis	Sprague Dawley

**FIGURE 6:**
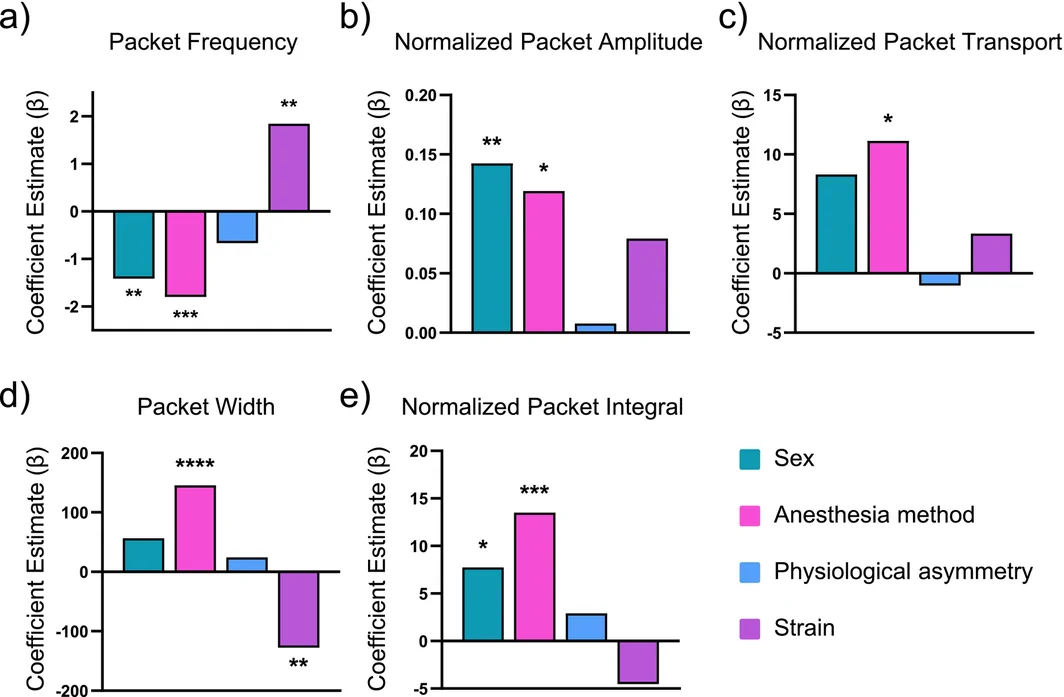
Linear mixed‐effects model used to assess the influence of sex, anesthesia method, physiological asymmetry, and strain on in vivo lymphatic pumping metrics. Fixed‐effects coefficient estimates (β) are shown for (a) packet frequency, (b) normalized packet amplitude, (c) normalized packet transport, (d) packet width, and (e) normalized packet integral. Fixed effects included sex (green), anesthesia method (pink), physiological asymmetry (blue), and rat strain (purple). Asterisks denote significance levels: * = *p* < 0.05, ** = *p* < 0.01, *** = *p* < 0.001, and **** = *p* < 0.0001.

The linear mixed‐effects model revealed distinct influences of biological and experimental factors on lymphatic pumping metrics (Figure 6). For packet frequency, Sprague Dawley rats exhibited higher contraction rates compared to Lewis rats, while isoflurane anesthesia and male sex were associated with reduced frequency. Packet amplitude was greater in male rats and under ketamine/dexdomitor anesthesia, indicating stronger contractions in those conditions. Similarly, packet transport was elevated with ketamine/dexdomitor administration, particularly in male rats, suggesting enhanced peak flow. Packet width, which reflects the duration of contraction‐induced flow, was increased under ketamine/dexdomitor and in Lewis rats, whereas Sprague Dawley rats demonstrated shorter contraction durations. Finally, packet integral, representing total lymph moved per contraction, was higher under ketamine/dexdomitor anesthesia and in Lewis rats, indicating greater transport capacity relative to isoflurane and Sprague Dawley counterparts. These findings underscore the significant and differential roles that sex, anesthesia method, and strain play in modulating lymphatic contractile function.

## 4 Discussion

Understanding the physiological and experimental variables that influence lymphatic pumping is essential for developing standardized and reproducible animal models of lymphatic function and injury, as well as interpreting the effects of the variables when synthesizing results across the literature when different variations are used. In this study, we examined how sex, anesthesia method, physiological asymmetry, and rat strain affect in vivo CLV contractility and transport, using NIR imaging in the rat popliteal region.

Our results demonstrate that both sex and anesthesia method significantly modulate lymphatic function. Specifically, female rats showed reduced packet amplitude, transport, and integral but increased contraction frequency compared to males, suggesting sex‐specific regulation of lymphatic output and align with studies that have shown how chromosomal and sex hormones can influence a plethora of physiological functions, including lymphatic function [16, 47, 48]. Anesthesia method had similarly pronounced effects: rats administered with ketamine/dexdomitor exhibited higher contractile amplitude, transport, and integral compared to those under isoflurane. In contrast, isoflurane was associated with shorter and less forceful contractions. It has been shown previously that different methods of anesthesia can yield varying physiological effects systematically [10, 49]. This difference is likely attributed to differing neural components the form of anesthesia is activating. That is, ketamine activates the cortical nuclei, whereas isoflurane activates the hypothalamus and brain stem nuclei, which in turn yield different downstream physiological function [49]. Our findings contrast with those of a similar anesthesia study in mice, which reported that isoflurane reduced lymphatic contractility while ketamine/dexdomitor had no effect [25]. This discrepancy may be attributed to species‐specific physiological differences, as well as potential genetic variability in outbred mouse populations that could influence lymphatic responsiveness to anesthetics.

We also examined the influence of physiological asymmetry by comparing lymphatic function between left and right hindlimbs. Lymph from the upper right quadrant of the body drains from the right lymphatic duct into the right subclavian vein, while the entirety of remaining lymph from the rest of the body drains from the thoracic duct into the left subclavian vein [11, 12]. Despite the known anatomical differences in lymphatic drainage between body quadrants, we observed no significant differences in pumping metrics across limbs when controlling sex and anesthesia method. This suggests that regional symmetry of lymphatic function is preserved in the distal popliteal vessels, possibly due to their distance from the central anatomical divergence at the thoracic duct [50]. Additionally, we found that rat strain impacts lymphatic behavior. These differences may stem from underlying genetic variation in lymphatic LMCs, valve function, or endothelial signaling pathways.

Our findings also underscore the importance of considering the close functional coupling between the lymphatic and blood vascular systems when interpreting differences in lymphatic transport. Although this study focused on lymphatic contractility, the observed effects of sex, anesthesia, and rat strain may also be indirectly influenced by alterations in blood vascular physiology. Sex hormones regulate endothelial nitric oxide signaling, sympathetic activity, vascular tone, and microvascular permeability, all of which can affect capillary filtration and the rate of lymph formation. Anesthetic agents modify systemic blood pressure, heart rate, autonomic activity, vascular resistance, and tissue perfusion, indicating that anesthesia‐dependent differences in lymphatic pumping may reflect both direct effects on lymphatic vessels and changes in lymphatic preload and afterload due to altered fluid filtration. Additionally, rat strains can exhibit differences in vascular reactivity, endothelial function, inflammatory phenotype, and microvascular permeability, which may further influence interstitial fluid production and lymphatic loading. Because blood vascular parameters were not measured concurrently in this study, it is not possible to determine the extent to which the observed differences in lymphatic transport were mediated by changes in the blood vasculature compared to intrinsic lymphatic mechanisms.

While these findings contribute valuable insight, several limitations should be acknowledged. First, all experiments in this study were conducted solely in the popliteal region with NIR imaging. It has been shown that vessel architecture, flow patterns, and contractility differ in other lymphatic beds, such as the tail and mesentery [44, 51, 52, 53]. Furthermore, it is important to note that capturing lymphatic function with NIR imaging is limited to superficial lymphatics in the periphery. There is currently no imaging solution available that allows for non‐invasive functional lymphatic imaging of the deeper lymphatics, where many of the regional functional differences in isolated vessel studies have been observed. Second, all clinical NIR imaging is performed without anesthesia, a practice that is challenging to translate to animal research due to multiple technical hurdles [54, 55, 56]. Because all imaging in this study was performed under anesthesia, it is likely that lymphatic function in the awake state may differ substantially. Prior studies have shown that anesthesia can suppress lymphatic function to baseline by inhibiting various sympathetic and parasympathetic processes [25, 57].

In conclusion, this study demonstrates that sex, anesthesia method, and strain are critical variables that influence lymphatic pumping behavior and must be carefully considered when designing and interpreting lymphatic research. These findings help establish a foundation for a standardized rat model of lymphatic function and injury, which is crucial for improving reproducibility across preclinical studies and for advancing translational strategies to treat lymphatic injury.

## 5 Perspectives

Investigating the effect of anesthesia method, sex, and lymphatic physiological asymmetry on lymphatic vessel contractility and transport helps to solidify the specifications of a standardized rat lymphedema/lymphatic injury model. Accounting for these variables may improve experimental reproducibility and the comparison of lymphatic outcomes across studies.

## Author Contributions

Rudolph L. Gleason, J. Brandon Dixon, and Zhanna Nepiyushchikh designed and conceptualized the study. Rachel Chin and Zhanna Nepiyushchikh conducted experiments, while Sophia Mavris and Zhanna Nepiyushchikh analyzed and interpreted the data. Sophia Mavris wrote the manuscript, and Rudolph L. Gleason, J. Brandon Dixon, and Zhanna Nepiyushchikh edited the manuscript.

## Funding

National Institutes of Health grant R01‐HL152152.

## Ethics Statement

All protocols involving animals conformed to the National Institutes of Health Guide for the Care and Use of Laboratory Animals and were approved by the Georgia Institute of Technology Animal Care Facility.

## Conflicts of Interest

The authors declare no conflicts of interest.

## Data Availability

The data presented in this study are available upon request from the corresponding author, Zhanna Nepiyushchikh.
